# Drug Integrating Amphiphilic Nano-Assemblies: 2. Spatiotemporal Distribution within Inflammation Sites

**DOI:** 10.3390/pharmaceutics16050652

**Published:** 2024-05-13

**Authors:** Teresa De Toni, Teodora Dal Buono, Chris M. Li, Grisell C. Gonzalez, Sung-Ting Chuang, Peter Buchwald, Alice A. Tomei, Diana Velluto

**Affiliations:** 1 Diabetes Research Institute, Miller School of Medicine, University of Miami, Miami, FL 33136, USA; detoniteresa2@gmail.com (T.D.T.); txd1299@miami.edu (T.D.B.); cml229@med.miami.edu (C.M.L.); g.gonzalez29@umiami.edu (G.C.G.); sxc2158@med.miami.edu (S.-T.C.); pbuchwald@med.miami.edu (P.B.); atomei@med.miami.edu (A.A.T.); 2 Department of Biomedical Engineering, University of Miami, Miami, FL 33146, USA; 3 Department of Microbiology and Immunology, Miller School of Medicine, University of Miami, Miami, FL 33136, USA; 4 Department of Molecular and Cellular Pharmacology, Miller School of Medicine, University of Miami, Miami, FL 33136, USA; 5 Department of Surgery, Miller School of Medicine, University of Miami, Miami, FL 33136, USA

**Keywords:** block-copolymers, self-assembling, nanoparticles, drug delivery, local immunomodulation, cell transplantation

## Abstract

The need for chronic systemic immunosuppression, which is associated with unavoidable side-effects, greatly limits the applicability of allogeneic cell transplantation for regenerative medicine applications including pancreatic islet cell transplantation to restore insulin production in type 1 diabetes (T1D). Cell transplantation in confined sites enables the localized delivery of anti-inflammatory and immunomodulatory drugs to prevent graft loss by innate and adaptive immunity, providing an opportunity to achieve local effects while minimizing unwanted systemic side effects. Nanoparticles can provide the means to achieve the needed localized and sustained drug delivery either by graft targeting or co-implantation. Here, we evaluated the potential of our versatile platform of drug-integrating amphiphilic nanomaterial assemblies (DIANAs) for targeted drug delivery to an inflamed site model relevant for islet transplantation. We tested either passive targeting of intravenous administered spherical nanomicelles (nMIC; 20–25 nm diameter) or co-implantation of elongated nanofibrils (nFIB; 5 nm diameter and >1 μm length). To assess the ability of nMIC and nFIB to target an inflamed graft site, we used a lipophilic fluorescent cargo (DiD and DiR) and evaluated the in vivo biodistribution and cellular uptake in the graft site and other organs, including draining and non-draining lymph nodes, after systemic administration (nMIC) and/or graft co-transplantation (nFIB) in mice. Localized inflammation was generated either by using an LPS injection or by using biomaterial-coated islet-like bead implantation in the subcutaneous site. A cell transplant inflammation model was used as well to test nMIC- and nFIB-targeted biodistribution. We found that nMIC can reach the inflamed site after systemic administration, while nFIB remains localized for several days after co-implantation. We confirmed that DIANAs are taken up by different immune cell populations responsible for graft inflammation. Therefore, DIANA is a useful approach for targeted and/or localized delivery of immunomodulatory drugs to decrease innate and adaptive immune responses that cause graft loss after transplantation of therapeutic cells.

## 1. Introduction

The nanomaterial revolution has its scientific foundations in 1959, when the physicist Richard Feynman suggested that it would be possible to manipulate matter at level of individual atoms [1]. Since then, there have been several scientific breakthroughs resulting in countless scientific papers being published, many products placed on the market, and three Nobel prizes awarded for work in nanoscience and nanotechnology. There are now >30 nanoparticle therapies and diagnostics approved for clinical use [2,3,4,5], and some of the most innovative solutions have been implemented in drug delivery. A drug delivery system is a formulation, device, or carrier that enables the introduction of a therapeutic substance, e.g., an immunosuppressant, in the body while improving its efficacy and safety by controlling the rate, time, and place of its release [6]. Such carriers are typically designed to transport drugs more precisely to their pharmacological target, away from sites of toxicity, and/or to maintain drugs at a therapeutic concentration over longer periods of time [7]. Ideal carriers must be biocompatible, biodegradable, water friendly, selective, easy to prepare, stable, cheap, and, finally, ultra-small. Therefore, nanoparticles with dimensions smaller than approximately 100 nm can play an important role in drug delivery by providing drug loading, solubilizing poorly water-soluble agents [8,9], protecting from degradation caused by endogenous mechanisms, reducing dosage and toxicity, and increasing therapeutic efficacy. They can also provide sustained and localized drug delivery to specific cells or tissues by exploiting either a site-specific stimuli (pH, temperature, light, or reduction/oxidation) as a release trigger [10] or modified morphology/size that allows for passive targeting.

Liposomes and lipid nanoparticles are currently the nanoscale systems most used for drug and gene delivery [11], but also quantum dots, gold nanoparticles, carbon nanotubes, dendrimers, nanogels, and biodegradable polymers have found valuable applications in the biomedical field. In particular, biodegradable polymers are highly promising [12] and some are already FDA approved for clinical use [13]. Many drugs, such as anticancer drugs, nucleic acid-based drugs, and immunomodulatory drugs, although very potent, suffer from poor stability and water solubility, low bioavailability, and narrow therapeutic window.

**Scheme 1:**
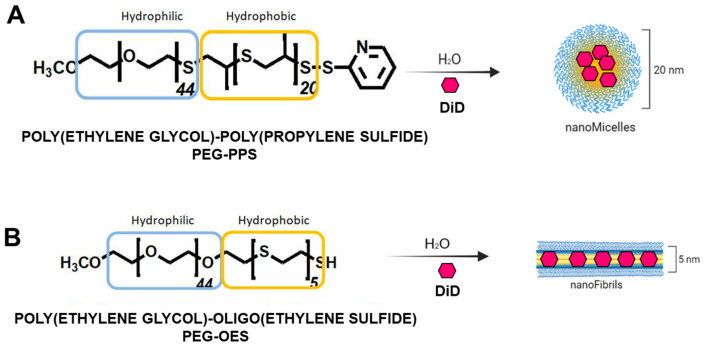
Chemical composition of PEG_44_-PPS_20_ (**A**) and PEG_44_-OES_5_ (**B**) diblock-copolymers, and self-assembling into nanomicelles (nMIC) and nanofibrils (nFIB) with a cargo dye encapsulation.

Building on this background and aiming to improve the pharmacological properties of existing drugs, we previously developed a versatile, biocompatible, and low-cost platform of therapeutic nanoparticles named drug-integrating amphiphilic nanomaterial assemblies (DIANA) [14]. DIANAs are fabricated in-house starting from the synthesis of amphiphilic di-block copolymers that belong either to the poly(ethylene glycol)-poly(propylene sulfide) (PEG-PPS) or the poly(ethylene glycol)-oligo(ethylene sulfide) (PEG-OES) families (Scheme 1) [9,14]. Each block copolymer can self-assemble in water, forming a variety of stable nanostructures whose size and morphology are determined by the amounts of hydrophilic (PEG) and hydrophobic (PPS or OES) components present [15,16]. They include nanomicelles, nanofibrils, and polymersomes. These nanostructures form spontaneously at very low critical aggregation concentration, which confers stability in vivo, mostly in blood circulation. Because DIANA nanoparticles are obtained from our own custom-synthesized amphiphilic block-copolymers, they can be designed to have unique biocompatibility and biodegradability as well as controllable hydrophobicity, size, and predictable aggregation morphology [15,16] that tune drug loading efficiency and targetability. Moreover, our synthetic method for preparing DIANA amphiphilic block copolymers is based on in-situ-generated thiolates that ensure propagation but also control the block lengths (reproducibility). In addition, thiolated polymers are mild-character reactive species that allows to easily incorporate even sensitive bioactive groups (e.g., peptides and small nucleotides) into the polymer backbone, before they self-aggregate [9,17].

Among the different PEG-PPS and PEG-OES block compositions, we have recently focused on the PEG_44_PPS_20_ and PEG_44_OES_5_ block copolymers because they showed a distinct lyotropic behavior, building homogeneous anisotropic structures when put in contact with water, as well as efficient drug loading and retention [14]. These di-block-copolymers self-assemble in water, forming biocompatible nanomicelles (nMIC, ~20 nm diameter) and nanofibrils (nFIB, ~5 nm diameter/1 μm length), respectively (Scheme 1). Furthermore, due to the nature of the PPS and OES blocks, this platform is particularly useful to solubilize and stabilize hydrophobic drugs, including potent immunomodulatory agents [9,14]. Both nMIC and nFIB have been shown to provide one of the highest water solubilities reported for cyclosporine A (CsA; 4.5 mg/mL) together with two-week sustained release in vitro, efficient uptake into immune cells, morphology-controllable biodistribution following subcutaneous administration, and effective immune suppression at lower dosage than that used with unformulated CsA [14]. Therefore, we believe that our nanomaterial platform could significantly improve many current treatments, particularly those chronic treatments needed for transplantation of cells and tissues like β-cell replacement therapies in patients with type 1 diabetes (T1D) [18,19] to prolong graft survival and function while minimizing unwanted deleterious side effects [20,21].

Towards this aim, we sought to use DIANA nanoparticles for the development of immunomodulatory therapies that are localized in the vicinity of transplanted cells to restrain anti-inflammatory and immune suppressive therapies that can prevent cell rejection and minimize adverse effects in the rest of the body. To do this, nanoparticles must be able to target and deliver their cargo at the site of an acute inflammation. Our previous biodistribution studies after subcutaneous delivery of nMIC and nFIB in mice indicated that nFIB remain more localized, reaching only lymph nodes draining the injection site, whereas nMIC can also target distal lymph nodes [14]. However, the effect of size and shape of DIANA nanoparticles on their systemic biodistribution, which strongly influence their therapeutic effects and toxicity, has not been evaluated yet. The chemical and physical properties of the nanoparticles, such as size, shape, surface charge, and surface chemistry, are important factors that determine the pharmacokinetics and biodistribution of their cargo [22]. Finally, the intracellular fate of the nanoparticles after cellular internalization also affects drug bioavailability and specificity justifying an analysis of the cellular uptake.

We believe that the application of nMIC and/or nFIB for targeted and localized inflammatory and immune modulation should be beneficial for allowing long-term functionality of transplanted cells while minimizing systemic toxicity of immunomodulatory drugs. This approach is particularly well suited for insulin-producing cell transplantation in patients with T1D, where the cells are implanted in well-defined and confined spaces [23] making targeted and sustained release more feasible. Despite the substantial evolution in the field of pancreatic β-cell replacement therapies over the last decades [24,25,26,27], this procedure is still associated with uncontrolled inflammation, allo-rejection, and recurrence of autoimmunity. When an inflammation occurs, the walls of blood vessels around the inflamed area/tissue become ‘‘leaky’’, allowing molecules and small particles to extravasate [28,29]. Drug molecules can diffuse through this breach of the vessels and escape cellular endocytosis, while the extravasation of nanoscale drug delivery vehicles through the leaky vasculature causes subsequent inflammatory cell-mediated sequestration. This process can be exploited as a passive targeting mechanism [28,29] and allows nanoparticles to accumulate preferentially in the inflamed site carrying their drug payload, thus achieving enhanced anti-inflammatory efficacy at the site of interest.

Here, we present a comprehensive evaluation of the biodistribution of DIANAs in the presence of an inflammatory signal modeling the inflamed cell graft site. To do this, nMIC and nFIB were loaded with a fluorescent lipophilic cargo probe that is known to be stably retained in the hydrophobic cores of the DIANAs [14]. Our goal is to demonstrate that DIANA nanoparticles can provide effective passive targeting to inflamed tissues, such as transplant sites, and upon arrival, can be retained locally for a clinically relevant amount of time and taken up by the recruited immune cells. To simulate a target site, we induced acute inflammation in mice by either local injection of lipopolysaccharide (LPS) or implantation of biomaterial-coated beads (CB). The nMIC and nFIB uptake by different immune cell subtypes playing a role in cell transplant rejection, including lymphocytes, macrophages, and granulocytes was also evaluated. Finally, we assessed the biodistribution of systemically administered nMIC and co-implanted nFIB in mice that received a transplant of pancreatic islets in a confined extrahepatic space.

Overall, we show that nanoparticle shape causes significant differences in internalization, circulation times, stability, and cytotoxicity with contrasts between spherical and fibrillar nanoparticles highlighted here. Nanofibrils of PEG_44_OES_5_ with a length > 1 μm and a cross-sectional diameter of only a few nanometers possess a large surface area that allows for more efficient cell contact and internalization in vitro [30] than spherical nanoparticles, as well as slower circulation in vivo due to decreased sensitivity to flow forces [31]. Therefore, we intended to prove that our nanofibrils can be used for localized accumulation in the areas closer to the administration site. On the other hand, spherical PEG_44_PPS_20_ nanomicelles with diameters of 20–25 nm can load larger amounts of hydrophobic cargos than the nanofibrils and provide more sustained release [9,14]. Here, we intended to show that nanomicelles can circulate in vivo in the blood stream and accumulate at sites distal from the site of administration and into poorly accessible/vascularized organs. Synergistic uses of nMIC and nFIB could provide significant improvement in the drug delivery field.

## 2. Materials and Methods

Commercial grade reagents and HPLC-grade solvents were purchased from VWR (Radnor, PA, USA) and Sigma-Aldrich (St. Louis, MO, USA) and directly used without further purification. The far-red fluorescent, lipophilic carbocyanine DiD (1,1′-dioctadecyl-3,3,3′,3′-tetramethylindodicarbocyanine, 4-chlorobenzenesulfonate salt), and the NIR fluorescent, lipophilic carbocyanine DiR (1,1′-dioctadecyl-3,3,3′,3′-tetramethylindotricarbocyanine iodide) were obtained as solid compounds from Invitrogen (Waltham, MA, USA).

### 2.1. Synthesis of the PEG_44_PPS_20_ and PEG_44_OES_5_ Block Copolymers

Poly(ethylene glycol)-poly(propylene sulfide) (PEG_44_-PPS_20_) and poly(ethylene glycol)-oligo(ethylene sulfide) (PEG_44_-OES_5_) copolymers, obtained via anionic ring-opening polymerization of -PS (propylene sulfide) or -ES (ethylene sulfide) from a thiolated PEG macroinitiator, were synthesized as previously reported [9,14,15] and are briefly described as follows.

*Poly(ethylene glycol)-poly(propylene sulfide).* A linear monomethoxy-poly(ethylene glycol) (mPEG-OH, MW 2 kDa) was modified to obtain a thiol-protected group on the OH end of the chain (m-PEG-thioacetate); then, the thiol was activated in the presence of propylene sulfide (PS) to initiate the anionic ring-opening polymerization of 20 equivalents of this monomer. The chain terminus was reversibly capped by disulfide exchange with 2,2′ -dithiodipyridine to provide a PEG-PPS block copolymer that can be further functionalized, if necessary, by disulfide exchange reaction [32]. The obtained product was purified by precipitation in diethyl ether followed by vacuum filtration. The final compound was confirmed using 1H NMR spectroscopy performed in CDCl_3_ on a Bruker AVANCE (400 MHz) platform with Topspin software (version 4.4.0): *δ* = 1.35–1.45 (d, C**H**_3_ in PPS chain), 2.6–2.7 (m, -C**H** in PPS chain), 2.85–3.0 (m, -C**H**_2_ in PPS chain), 3.38 (s, -OC**H**_3_), 3.52–3.58 (t, -OC**H**_2_CH_2_S), 3.5–3.7 ppm (s, broad, -OC**H**_2_C**H**_2_ in PEG chain protons), 7.8–7.83 (m, 1**H**, pyridine group). The degree of polymerization of the PPS block was determined by the ratio of PEG to PPS protons.

*Oligo(ethylene glycol)-poly(propylene sulfide).* The same linear 2 kDa mPEG-OH was modified and activated, as for the PEG-PPS synthesis, to initiate the anionic ring-opening polymerization of 5 equivalents of ethylene sulfide that yields to the growth of a penta-(ethylene sulfide) oligomer from the PEG terminus. The reaction was terminated with excess of glacial acetic acid, and the product was purified by repeated precipitations in diethyl ether followed by vacuum filtration. The length and composition of the blocks were confirmed using 1H NMR spectroscopy (CDCl_3_) as before: *δ* = 3.5–3.7 (s, broad, -OC**H**_2_C**H**_2_), 3.38 (s, -OC**H**_3_), 2.87 (m, -C**H**_2_SH) 2.85 (m, -SC**H**_2_C**H**_2_), 2.74 (td, -C**H**_2_CH_2_SH). The degree of polymerization of the OES block was determined by the ratio of PEG protons to OES protons.

### 2.2. Preparation of Fluorescently Labeled Nanomicelles and Nanofibrils

Nanomicelles (nMIC) of 20 nm in diameter were obtained from a minimum of 20 mg to a maximum of 40 mg of PEG_44_-PPS_20_ block copolymer using the cosolvent evaporation method [9]. Simply, the PEG-PPS was dissolved in dichloromethane (0.5 mL) and then added dropwise to distilled water (1.0 mL). The mixture was stirred at room temperature and at open air until dichloromethane was completely removed by evaporation, at which point the aqueous phase contains nMIC. When needed, the complete evaporation of the organic phase was achieved under vacuum. Nanofibrils (nFIB) of 5 nm in diameter were obtained from a minimum of 40 mg to a maximum of 80 mg of PEG_44_-OES_5_ following the same procedure described above for the nMIC.

For the preparation of fluorescent PEG_44_-PPS_20_ nMIC and PEG_44_-OES_5_ nFIB, the protocol was modified to include a hydrophobic cargo probe into the PPS and OES nanoparticle cores. First, 10 mg of the DiD far-red lipophilic dialkylcarbocyanine dye were resuspended in dichloromethane to obtain a DiD stock solution of 2.5 mg/mL in concentration. Next, 250 μL (containing 0.625 mg) of the DiD solution was added to 40 mg of either PEG_44_-PPS_20_ or PEG_44_-OES_5_ block-copolymers that were previously dissolved in 250 μL of dichloromethane. The combined solution of 500 μL, containing one of the investigated block copolymers, and the DiD probe was added to 1.0 mL of distilled water dropwise to form an emulsion that was stirred at room temperature and at open air until the dichloromethane was completely removed by evaporation. The remaining aqueous phase contained the DiD-labeled nMIC (nMIC-DiD) or nFIB (nFIB-DiD), respectively. Both nMIC-DiD and nFIB-DiD were centrifuged at high speed and extensively dialyzed against 1000-fold their volume of deionized water (MWCO 12 kDa) to remove possible unloaded dye molecules. The purified stock solutions contained 0.625 mg/mL of the DiD loaded into either the nMIC or the nFIB, and were stored at 4 °C and used either in vitro diluted 100-fold or in vivo at different dilutions, as specified in the related Sections. In some experiments, DiR, an analog of DiD, was used instead. DiR has excitation and emission maxima in the near infrared region, where many tissues are optically transparent. Nanoparticle labeling procedures and dye concentration were the same as described above for the DiD probe.

### 2.3. In Vitro Cellular Uptake of nMIC-DiD and nFIB-DiD into Human Pancreatic Islets

Human islets (HIs) were procured from the current Good Manufacturing Practice Human Islet Cell Processing Facility (University of Miami, Miami, FL, USA). HIs were isolated using a modification of the Clinical Islet Transplant consortium’s standardized automated method and under an exemption issued by the University of Miami Institutional Review Board [33,34]. After the purification process, the islets were cultured overnight at 37 °C in untreated flasks and in complete media consisting of PIM R (Prodo Labs, Aliso Viejo, CA, USA; Cat# PIM R 001-GMP) supplemented with 2% PIM G (Prodo Cat# PIM G-001-GMP), 5% Human Serum AB (GeminiBio, West Sacramento, CA, USA; Cat# 100-512), and 5 mL of 2 mg/mL ciprofloxacin (Claris Lifesciences, Cupertino, CA, USA; Cat# 36000-009-24). Islet tissue volume was measured by placing 100 μL samples of suspended islets into an optically clear dish (Biorep, Miami Lakes, FL, USA; Cat# ICC-D2), diluting with 200 μL PBS, and quantifying with an ICC4 automated counter (Biorep, Miami Lakes, FL, USA) reporting islet equivalents (IEQ) [35]. Aliquots of 150 IEQ were suspended in fresh complete media and placed on gas-permeable culture plates (Miltenyi Biotec; Waltham, MA, USA) that have been designed for high density cell cultures. Each aliquot was treated with either nMIC-DiD or nFIB-DiD, diluted 100-fold from stock, and incubated for 24 h. At the end of the incubation, islets were extensively washed with PBS, incubated for 30 min with 20 μM Hoechst (Invitrogen; Waltham, MA, USA) in complete media to stain the cell nuclei, washed and resuspended in fresh PBS, and imaged with a Leica (Wetzlar, Germany) DMi8 optical microscope.

### 2.4. Mice

All studies involving animal subjects were performed under protocols approved and monitored by the University of Miami (UM) Institutional Animal Care and Use Committee (IACUC protocols 22-136, 22-013). All procedures were conducted according to the guidelines of the Committee on Care and Use of Laboratory Animals, Institute of Laboratory Animal Resources (National Research Council, Washington, DC, USA). All animals were obtained from Jackson Laboratories (Bar Harbor, ME, USA) and housed at the Division of Veterinary Resources, University of Miami.

### 2.5. Passive Targeting and/or Localized Delivery of DIANAs into a Site of Acute Inflammation

To investigate the spatio-temporal distribution of nMIC and nFIB DIANAs and their cargos after systemic administration, three different animal models bearing an inflammatory condition were used here and are described below.

#### 2.5.1. Model 1: Subcutaneous Injection of LPS in Mice

A simple model of localized acute inflammation was created using subcutaneous injection of 25 μL of 1 mg/mL lipopolysaccharide (LPS, Sigma-Aldrich, 25 μg total dose) in saline in the right foot paw of SKH-1 or BALB/c mice (males, 10–12 weeks old obtained from Jackson Laboratories; Bar Harbor, ME, USA). Either immediately or a few hours later, 50 μL of fluorescent nMIC-DiD or nFIB-DiD from the stock solutions (prepared as described above) was administered intravenously via the mouse tail vein. An optical in vivo imaging system (IVIS, PerkinElmer, Waltham, MA, USA) configured to detect DiD emission was used to evaluate the spatiotemporal accumulation of the fluorescent nMIC and nFIB into the site of the LPS-induced inflammation. The whole-body imaging was performed 1, 4, and 6 days after the nanoparticle administration. Untreated mice were imaged as negative control. The fluorescent intensity was quantified with the region of interest (ROI) method using the Living Imaging Software (version 4.7.3; PerkinElmer, Waltham, MA, USA) that accompanies the IVIS spectrum system. The ROI intensity of the right foot paw of each treated mouse was compared to the ROI of the left foot paw (contralateral site). At day 7 after nanoparticle administration, mice were euthanized, and major organs of interest (liver, spleen, lungs, pancreas, and kidneys) were extracted and imaged with an IVIS spectrum to evaluate any DiD fluorescence signal due to nMIC-DiD or nFIB-DiD off-targeting.

#### 2.5.2. Model 2: Subcutaneous Implantation of PEG Coated Polystyrene Beads in Mice

*(i) Polystyrene bead encapsulation*

Polystyrene beads with islet size distribution mimicking that of primary human islets were prepared by mixing 3.06, 17.24, 38.11, and 41.57 mg of 50, 100, 150, and 200 µm diameter Red ChromoSphere beads (ThermoFisher Scientific, Waltham, MA, USA), respectively, as previously reported. Bead mixtures were suspended in 5% Bovine Serum Albumin (BSA; Sigma-Aldrich, St. Louis, MO, USA) in PBS, washed with HBSS-/- buffer (ThermoFisher Scientific, Waltham, MA, USA), and coated with a PEG hydrogel biomaterial, as previously reported [36]. For this, 6.05% (*w*/*v*) 10 kDa 8-arm 75% functionalized PEG-maleimide (PEG-MAL; JenKem Technology, Plano, TX, USA) was partially crosslinked with 36.2% (*w*/*v*) 2kDa PEG-dithiol (HS-PEG2000-SH; JenKem Technology, Plano, TX, USA). Coated beads (CB) were resuspended in this viscous solution and extruded through a proprietary microfluidic device, produced in conjunction with Biorep (Miami, FL, USA). A 10% Span80 (Sigma-Aldrich, St. Louis, MO, USA) in polypropylene glycol (Sigma-Aldrich, St. Louis, MO, USA) was used as an oil carrier solution. A separate solution of 25 mg/mL dithiothreitol (VWR, Radnor, PA, USA) in polypropylene glycol with 10% Span80 was flowed coaxially along the outlet of the device to provide reducing conditions and complete polymer cross-linking after extrusion. CBs were then incubated in complete human islet media for 48 h. CBs were quantified in IEQ using the same process as for human islets described above, but diameters were measured manually using a Leica (Wetzlar, Germany) DMIL light microscope.

*(ii) Subcutaneous bead implantation procedure in mice*

C57BL/6J mice or SKH-1 hairless mice were used as recipients of coated beads (CB), which was performed as described previously [36,37]. Briefly, mice were anesthetized with 2% isoflurane, and either the left or the right inguinal area and medial hindlimb were shaved and sanitized with an alcohol towelette. A 0.25 cm vertical incision was made in the inguinal area, and a pocket was made in the underlying subcutaneous white adipose tissue superior to the inferior epigastric artery. Coated beads (750 IEQ) were resuspended in 10 μL of sex- and strain-matched plasma and implanted into the subcutaneous (SC) pocket. 5 μL of recombinant thrombin (Recothrom, Zymogenetics, Seattle, WA) was added on top of the CB-plasma mixture to form a gel-like biologic scaffold (BSc) [23]. As a positive control of local inflammation, 25 μL of 1 mg/mL LPS in saline were added in the BSc implanted SC in the left inguinal area.

*(iii) Spatiotemporal distribution of nMIC-DiD and nFIB-DiD in the implant site*

To test the localization and retention of nFIB within the subcutaneous implant site (SC), 5 µL of stock concentration nFIB-DiD generated as described above was added directly into the mixture containing coated beads or LPS and autologous plasma prior to implantation. On the other hand, to test the passive targeting ability of nMIC to the SC graft site, 50 μL of the nMIC-DiD was administered via intravenous infusion once on the day of the CB implantation. The same amounts of nMIC-DiD were also administered into mice bearing an implantation containing LPS as a positive control of local inflammation, and into mice without any implantation.

To track and quantify the in vivo distribution of both fluorescent DIANAs, whole-body images of mice were taken from day 1 up to 21 or 22 days after implantation using the IVIS spectrum configured to detect the emission of DiD. Untreated mice were imaged as a negative control and used to remove fluorescent background. nMIC-DiD and nFIB-DiD fluorescence intensity was quantified with the Living Image software using the inguinal area as region of interest (ROI). Distribution of fluorescent DIANAs was also determined ex vivo, where at days 1 and 21 or 22, mice were euthanized, and the following organs were explanted: SC implant site (containing either CB or LPS within a BSc), inguinal lymph node (ipsilateral LN), brachial lymph node (contralateral LN), heart, lungs, liver, pancreas, spleen, and kidneys. Explanted organs were placed into 10 cm Petri dishes (Corning, Corning, NY, USA) and imaged using the IVIS spectrum system. Fluorescence intensity was quantified using the Living Imaging Software and the ROI method.

*(iv) nMIC-DiD and nFIB-DiD* in vivo *cellular uptake*

Immediately after acquiring the ex vivo images with the IVIS spectrum, the above explanted organs (on days 1 and 21 or 22) were placed on ice. Tissues were then transferred to a 40 μm cell-strainer, placed upon a 50 mL tube, and mechanically disrupted using the plunger of a 3 mL syringe to generate single immune cell suspensions for flow cytometry. Cell suspensions were re-filtered through a fresh 40 μm strainer, centrifuged at 450 RCF for 5 min at 4 °C, and treated with ACK (Ammonium-Chloride-Potassium) lysis buffer (ThermoFisher, Cat# A1049201) for 4 min to remove red blood cells. Isolated cells were stained with a Live/Dead Fixable Near-IR Cell Stain Kit (ThermoFisher, Cat# L10119, dilution 1:1000) and with antibodies against the following surface markers: anti-mouse CD3 eF450 (ThermoFisher, Cat# 48-0032-82, dilution 1:100), anti-mouse CD45 BV510 (Biolegend, Cat# 103137, dilution 1:1000), anti-mouse CD11b BV650 (Biolegend, Cat# 101259, dilution 1:100), anti-mouse CD11c BV786 (BD, Cat# 563735, dilution 1:500), anti-mouse F4/80 FITC (eBioscience, Cat# 11-4801-82, dilution 1:200), anti-mouse MHC II PE (eBioscience, Cat# 553548, dilution 1:1000), anti-mouse CD19 PE-CF594 (BD, Cat# 562291, dilution 1:200), anti-mouse CD206 PE-Cy7 (ThermoFisher, Cat# 25-2061-82, dilution 1:320), and anti-mouse Ly6G Alexa Fluor 700 (Biolegend, Cat# 1127613, dilution 1:400). Specific cell subpopulations were identified from live cells based on surface marker expression: T cells (CD3^+^), B cells (CD19^+^), and myeloid cells (CD3^−^CD19^−^CD11b^+^). From myeloid cells: dendritic cells (F4/80^−^CD11c^+^), neutrophils (F4/80^−^Ly6G^+^), M1-like macrophages (M1) (F4/80^+^MHCII^+^CD206^−^), and M2-like macrophages (M2) (F4/80^+^CD206^+^). The in vivo cellular distribution of DIANAs, either after IV administration or localized in the site of implantation, was also determined using flow cytometry quantification of the DiD fluorescence intensity within each of the above cell subpopulations. Data were obtained using a CytoFlex S flow cytometer and analyzed in Kaluza (Beckman Coulter, Brea, CA, USA).

#### 2.5.3. Model 3: Pancreatic Islet Cell Transplantation in Mice

*(i) nFIB-DiD localized distribution into syngeneic pancreatic islet transplantations*

A syngeneic transplant model (donors and recipients of the same species and strain) was adopted for this experiment because it does not suffer of immune attacks and immune rejection. Therefore, C57BL/6 mice (males, 10–12 weeks old), rendered diabetic via intraperitoneal injection of streptozotocin (STZ) [23], were implanted in the epididymal fat pad (EFP) with 600 IEQ isolated from healthy C57BL/6 mice. Diabetes was confirmed by three consecutive blood glucose readings > 350 mg/dL. To test the localization and retention of nFIB in the graft, 15 μL of nFIB-DiR (prepared as described above) were added in the fat pad in the closest vicinity of the islets. Briefly, under isoflurane anesthesia, a 1 cm lower abdominal midline incision allowed for the extraction of the left epididymal fat pad (EFP) that was spread flat over a sterile field. Islets and nFIB-DiR were immobilized on the EFP using a BSc. The fat pad was then folded over the islets, sealed with additional BSc, placed back to the abdominal cavity, and the incision closed with a 5-0 absorbable suture (Ethicon, Cincinnati, OH, USA). The spatiotemporal localization of nFIB in the site of islet implantation was monitored at post-operative days 3, 4, 5, and 7 via IVIS spectrum configured to detect the near infrared DiR fluorescence emission. Mice implanted only with islets were used as negative controls. Islet transplant efficacy was also determined by monitoring the capacity to achieve and maintain normo-glycemia (non-fasting blood glucose level < 250 mg/dL). At days 7 and 14, selected mice were euthanized and the EFP were extracted and imaged ex vivo to confirm the presence of fluorescence nanoparticles within the tissue.

*(ii) nMIC-DiD targeted distribution into allogeneic pancreatic islet transplantations*

An allogeneic pancreatic islet transplantation model of major MHC mismatch between the islet donor mice and the recipients, resulting in acute graft rejection, was used for the purpose of causing acute inflammation and impairing the integrity of the wall of blood vessels around the inflamed tissue (the EFP graft site). Briefly, 750 IEQ islets isolated from DBA/2 mice (males 12–14 weeks old) were implanted into chemically induced (STZ) diabetic C57BL/6 mice in the left gonadal fat pad site using a BSc as described above. Specifically for this experiment, transplanted mice were administered with 50 μL of nMIC-DiD via the tail vein infusion on the day when rejection of the graft was confirmed (blood glucose readings > 250 mg/dL for at least three consecutive days). The ability of 20 nm nMIC nanocarriers to circulate in the blood and passively target the graft site via an enhanced permeability effect was evaluated using an IVIS spectrum. Whole-body images of transplanted diabetic mice were taken on day 1 and 4 after the nMIC-DiD administration, and the ROI method was applied to measure the emission of the left lower abdominal area in correspondence to the implanted left EFP. At the same time points (day 1 and day 4), and immediately after the in vivo whole-body imaging, mice were euthanized and the left EFP containing the transplant, the right contralateral EFP, and the major organs (liver, spleen, lungs, and kidneys) were extracted and imaged ex vivo with the IVIS spectrum. The fluorescence emission of each organ was normalized for its corresponding mass weight (mg), and the resulting values were determined where the nMIC-DiD preferentially accumulated independently from the size of the tissue.

### 2.6. Statistics

Data were plotted in GraphPad Prism (GraphPad, La Jolla, CA, USA) and analyzed by Student’s t-test and one- or two-way analysis of variance (ANOVA) followed by Tukey’s post hoc multiple comparison test. Tests were modified to account for repeated measures where appropriate. Data were checked for normality of residuals and homoscedasticity, and tests were substituted with nonparametric equivalents (Wilcoxon and Kruskal–Wallis with Mann–Whitney tests) when these conditions were not met. A value of *p* < 0.05 was considered statistically significant. Data are presented as mean ± standard deviation.

## 3. Results and Discussion

### 3.1. Synthesis of the PEG_44_PPS_20_ and PEG_44_OES_5_ Block Copolymers and DiD-Labeled nMIC and nFIB

The PEG_44_PPS_20_ and PEG_44_OES_5_ block copolymers have been already used by our group for drug delivery applications. The design, the synthesis, the characterization of their chemical composition, their self-assembling properties, and their morphological analysis have been extensively described in previous publications. The loading efficiency of different molecules has been also reported by us, including the loading and stability of lipophilic fluorescent molecules such as those used in this work (DiD and DiR). Therefore, for what concerns this section, readers can find details in the cited references [9,14,15].

### 3.2. In Vitro Uptake of nMIC-DiD and nFIB-DiD into Human Islets

**Figure 1:**
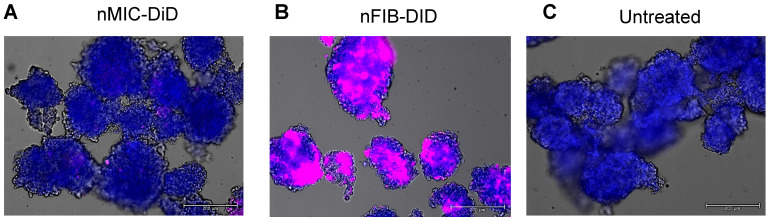
In vitro uptake of nMIC and nFIB DIANAs into human islet cells. Optical fluorescent microscope images of human islets treated with nMIC-DiD or nFIB-DiD. Aliquots of 150 islet equivalents (IEQ) were treated with either (**A**) nMIC-DiD or (**B**) nFIB-DiD 100-folds diluted from the stocks to a concentration of 6.25 μg/mL per aliquot and incubated for 24 h. (**C**) Untreated islets were used as control. Images are shown at 10× magnification with DiD in magenta, and Hoechst stained cells are shown in blue. Scale bars = 200 μm.

We already demonstrated that both nMIC-DiD and nFIB-DiD are taken up by different cells in vitro and by the immune cells residing in the lymph nodes draining the site of subcutaneous injection in vivo [14]. Here, we tested the applicability of our nanoparticles for localized drug delivery at the cellular graft by therapeutic cell modification before transplantation. As we already demonstrated that DIANA nanoparticles are not toxic to human islets [14], we used freshly isolated human pancreatic islets to demonstrate the ability of nMIC and nFIB to associate with islets (either penetrate the islet cells or aggregate on their surface) to act as a local drug depot at the transplantation site. Human islets were treated with the same amount of nMIC-DiD (Figure 1A) or nFIB-DiD (Figure 1B), and fluorescence microscope images revealed that the elongated nFIB-DiD are efficiently aggregated with the islet cells within 24 h, whereas the spherical nMIC-DiD nanoparticles seem to associate less efficiently with the islet structure. This differential behavior is likely due to the difference in the morphology of these nanostructures, with the elongated nFIB fibrils benefitting from their multiple contact points with the islets and smaller cross-sectional diameter (5 nm) than nanomicelles (20–25 nm). Furthermore, the presence of thiol groups on the OES polymer chains (Scheme 1B) promotes the interactions of the fibrils with cell membrane proteins. Therefore, the PEG-OES nanofibrils easily aggregate on the surface of the islets, as shown by the high fluorescence signal in the corresponding microscope image (Figure 1B, magenta). This is a property that will enable co-transplanting nFIB with islets to ensure prolonged localized delivery of drugs at the graft site for preventing post-operative acute inflammation that can compromise the graft using immunomodulatory drugs as cargo, and/or to enhance islet survival and function using drugs that promote islet health. On the other hand, nMIC, which do not efficiently enter the islets (Figure 1A), could be administered systemically and used to target the graft site during the onset of the inflammation, potentially through redosing at selected time points, by exploiting their passive targeting ability (see later).

### 3.3. In Vivo Biodistribution of nMIC-DiD and nFIB-DiD in Mouse Models of Localized Inflammation

The following experiments have been performed to prove our hypothesis that small (20–25 nm) spherical micelles such as nMIC can transit within the circulatory system and accumulate at the site of inflammation. Specifically, we hypothesized that nMIC can quickly and efficiently reach an inflammation site after intravenous infusion (IV), even when the site is confined and served by limited blood flow, whereas nFIB, whose elongated shape (>1 μm) only allows for slow movements in the blood circulation, can only reach areas close to the administration site. Thus, while nMIC are potentially useful for passive graft targeting after systemic administration, nFIB would be suitable for co-implantation directly in the graft site, where they can be retained without diffusing away, and both can be used for localized drug delivery with reduced systemic side effects.

#### 3.3.1. Model 1: Subcutaneous Injection of LPS in Mice

Acute inflammation is a problem in many medical situations, including the case of allogeneic cell transplantation for regenerative medicine. Pancreatic islet transplantation in patients with brittle T1D to restore endogenous glucose-stimulated insulin production is an example. In such a case, the use of most anti-inflammatory agents is limited by their poor solubility, the high dose needed, islet toxicity, and lack of graft specificity hampering the long-term function of transplanted islets. Therefore, efficient pharmacological therapies against pathological inflammatory responses should ideally be targeted and/or localized to the inflammation site to reduce dosage, administration frequency, and side effects.

Here, we produced acute inflammation in a confined site by administering a toxic dose of LPS (25 μg) into the subcutaneous (SC) space of the right foot paw (RFP) of a mouse. In a pilot study, we used hairless SKH-1 mice that were treated with IV infusion of either nMIC-DiD or nFIB-DiD via the tail vein. Then, we tracked the distribution of the fluorescent nanoparticles by whole-body imaging of live mice at 1, 3, 6, and 24 h after infusion. The collected images show that both nMIC and nFIB preferentially accumulate at the site of LPS-induced inflammation (Supporting Information, Figure S1A and Figure S1B, respectively), although, at 24 h post-infusion, the nMIC also accumulates in areas corresponding to major organs (such as the lungs). Interestingly, when the DiD probe is infused as free dye (dissolved in 20% DMSO in 5% dextrose solution), mice do not demonstrate any fluorescent signal, neither accumulating at the inflammation site nor in other tissue (Figure S1C). This confirms the ability of nMIC and nFIB to efficiently load, stabilize, and carry hydrophobic compounds; indeed, DiD fluorescent emission is detectable only when the dye is dispersed in a hydrophobic environment. These preliminary results suggest that our DIANA nanoparticles can transport their cargo molecule to the inflammation site (the RFP in this case) via passive targeting likely due to the enhanced vascular permeability effect.

**Figure 2:**
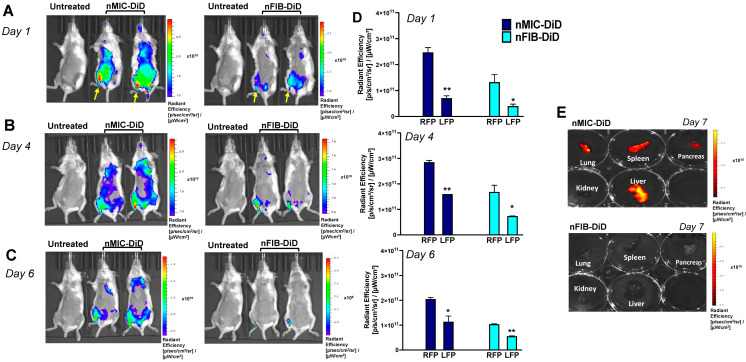
Biodistribution of nMIC and nFIB DIANAs in model 1 of acute inflammation as revealed by whole-body imaging. Localized acute inflammation was obtained using subcutaneous injection of 25 μL of LPS from 1 mg/mL solution in saline in the right foot paw (RFP) of BALB/c mice (yellow arrows). Inflamed mice (two per condition) were treated with nMIC-DiD or nFIB-DiD via IV infusion and analyzed using whole-body imaging with IVIS spectrum at day 1 (**A**), 4 (**B**), and 6 (**C**). Radiant efficiency color scales: min = 6.0 × 10^9^, max = 2.0 × 10^10^. (**D**) In vivo time-dependent DiD fluorescence intensity for the right- (ipsilateral) and left foot paws (LFP, contralateral) quantified using a Living Image Software with the region of interest (ROI) tool (baseline-normalized compared to the untreated mouse). Asterisks indicate statistically significant differences between the RFP and LFP (*n* = 2 independent animals): * *p* < 0.05, ** *p* < 0.01. (**E**) Ex vivo IVIS spectrum images of major organs (lungs, spleen, pancreas, kidneys, and liver) extracted on day 7 after infusion. Radiant efficiency color scales: min = 2.5 × 10^9^, max = 5.0 × 10^9^.

To strengthen these initial findings, similar experiments were performed using BALB/c mice (Figure 2). LPS (25 μg) was administered subcutaneously in the RFP of the mice (Figure 2A**,** yellow arrows). Two hours later, a period needed to ensure the onset of inflammation, animals received nMIC-DiD or nFIB-DiD via IV infusion in the tail vein. Live images of the animals were taken at day 1, 4, and 6 (Figure 2A, Figure 2B, and Figure 2C, respectively) after nMIC-DiD (left panels) or nFIB-DiD (right panels) administration to determine the passive targeting ability and the retention capability of these nanoparticles at the site of inflammation. IVIS imaging indicated accumulation of nMIC and nFIB at the inflammation site (RFP) after 24 h (Figure 2A) and retention through 6 days (Figure 2B,C). Additionally, while nMIC also diffuses into areas corresponding to the liver and lungs, the nFIB localized only in the RFP. The region of interest (ROI) quantification method was used to compare the fluorescent signals of the RFPs with the contralateral uninflamed site (the left foot paw, LFP) at each time point (Figure 2D). This analysis shows that the fluorescent intensity of either the nMIC- or the nFIB-DiD is significantly higher in the RFP with respect to the LFP, demonstrating that the DIANA nanoparticles, with distance and vascularization being equal, preferentially accumulate in the inflamed site. On day 7 after infusing the treatments, major organs of the peritoneal cavity were extracted and imaged with an IVIS spectrum ex vivo (Figure 2E). The images confirmed that nFIB did not significantly accumulate in those organs, but nMIC were abundantly present in at least the lungs, spleen, and liver, which are among the most vascularized organs [38]. Given their small size (20–25 nm) and spherical shape, the nMIC likely have a higher predisposition to escape circulation at highly vascularized sites and/or clearance by the mononuclear phagocyte/reticuloendothelial system [39].

Because the use of nMIC as delivery systems for hydrophobic immunomodulatory drugs is very attractive, in which they can load the drug efficiently and release it in a sustained manner, as we previously reported [9,14], we further investigated nMIC-DiD distribution in different inflammatory models, reported next. We hypothesized that the passive targeting ability of nMIC would be applicable to regenerative medicine for targeting inflamed sites such as the site of implantation of biomaterial-coated polystyrene beads (model 2) or of transplantation of allogeneic cells subject to immune rejection (model 3). Furthermore, we expected that the nFIB nanoparticles, because of their elongated shape that causes slower diffusion away from the site of administration, would be suitable for direct co-implantation within the graft site and enact localized sustained drug delivery.

#### 3.3.2. Model 2: Subcutaneous Implantation of PEG-Coated Polystyrene Beads in Mice

**Figure 3:**
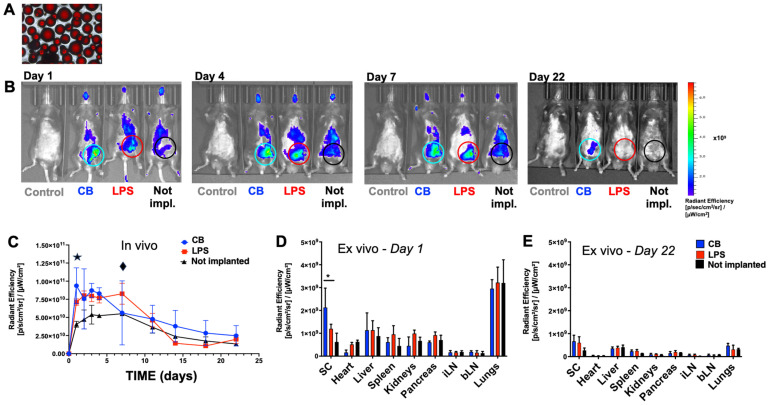
Biodistribution of nMIC-DiD in model 2 of acute inflammation. Localized acute inflammation was obtained using subcutaneous implantation (SC) of either PEG coated beads (CB, blue) or of 25 μL of LPS (red) into a biological scaffold (BSc). (**A**) Sample image of the polystyrene beads coated with crosslinked PEG-maleimide used. Magnification = 10×. (**B**) In vivo biodistribution observed via live optical whole-body imaging of C57BL/6 mice implanted with CB (blue circles), LPS (red circles), or not-implanted (Not impl.; black circles) and treated with IV infusion of 50 μL of nMIC-DiD. Selected time points are shown (1, 4, 7, and 22 days). Radiant efficiency color scales: min = 1.0 × 10^9^, max = 6.0 × 10^9^. (**C**) Time-profile of the nMIC-DiD fluorescence intensity quantified using the left inguinal area as region of interest (ROI) in mice with the CB implant (blue circles), LPS implant (red square), and no implant (black triangle) from day 1 to 22. (**D**,**E**) Distribution of nMIC-DiD determined ex vivo at day 1 (**D**) and 22 (**E**) in the SC implant site (containing either CB or LPS within a BSc), left inguinal lymph node (ipsilateral LN), right brachial lymph node (contralateral LN), heart, lungs, liver, pancreas, spleen, and kidneys. Explanted organs were imaged using the IVIS spectrum system and their fluorescence intensity was quantified using the Living Imaging Software and the ROI method. Data are expressed as mean +/− SD. A ★ symbol indicates significance between CB + nMIC-DiD and nMIC-DiD *p* < 0.05; ♦ symbol indicates significance between LPS + nMIC-DiD and MIC-DiD *p* < 0.05; * indicates significance between SC tissue compared to other tissues *p* < 0.05 (*n* = 3 independent animals).

Pancreatic islet cell encapsulation with permeable hydrogels has been studied for the past 30 years, with the goal of enabling transplantation without immunosuppression and restoration of insulin production in patients with T1D [40,41]. Despite considerable progress, clinical success has thus far been limited. Among possible reasons for the failure, the biocompatibility of the coating materials and the overall large surface area of coating biomaterials needed for surrounding the necessary number of transplanted cells for therapeutic efficacy trigger inflammation and fibrosis [42]. Furthermore, it has been suggested that the smaller the size of coating capsules, needed to limit graft volumes and transport of nutrients and therapeutic molecules (insulin), the stronger the inflammatory effect [42]. Covalently crosslinked eight-arm PEG hydrogels have been used by our group for coating islet cells to prevent their immunorejection in vivo, but localized anti-inflammatory modulation would be highly beneficial to enhance the biocompatibility of this coating [36]. Here, implantation of PEG hydrogel-coated polystyrene beads (CB) (Figure 3A) in subcutaneous adipose tissue was used not only to simulate therapeutic biomaterial transplantation, but also to induce acute inflammation in vivo.

*(i). Targeted distribution and cellular uptake of DiD-labeled nMIC*

Given their spherical morphology and our data (Figures S1 and S2), we assumed that nMIC could transit within the circulatory system more efficiently than nFIB, which have an elongated shape. Therefore, we tested the ability of nMIC to target the site of CB implantation after IV infusion. CBs (Figure 3A) with a size distribution resembling that of primary islets were implanted into the left inguinal subcutaneous white adipose tissue of C57BL/6J mice using a biologic scaffold (BSc) [23]. As a positive control for graft inflammation, 25 μg of LPS instead of CBs were added locally to the SC site. Mice that received an injection of nMIC-DiD but no implantation served as a negative control. nMIC-DiD were injected IV shortly prior to CB or LPS implantation, and their distribution was evaluated at selected timepoints between 1 and 22 days after administration. Panels in Figure 3B show live biodistribution of nMIC-DiD at selected time-points. As we expected, nMIC-DiD preferentially accumulated in the graft site containing the CBs (blue circle) as early as one day post-operation and up to day 7, similar to the graft containing only LPS (red circle). As anticipated, we did not observe targeted accumulation of nMIC-DiD in non-implanted mice. The intensity of nMIC-DiD was measured over time by ROI of the graft area and is expressed as total radiant efficiency (Figure 3C). The in vivo images and the ROI quantification together indicate that the nMIC-DiD fluorescent signal in the implantation site remains high through day 7, indicating that nMIC and their cargo are retained in the inflammation site for several days. The signal decreased progressively through day 22, but was still detectable at endpoint. We conclude that nMIC can be used for targeted delivery of therapeutics into a transplant site and for reducing administration frequency for maintaining local drug concentration in therapeutic range over time.

At days 1 and 22 post-transplantation, selected major organs were explanted and analyzed ex vivo using an IVIS spectrum. At day 1, nMIC-DiD signal accumulation in the CB implant site (Figure 3D, blue bars) was significantly higher than in mice that did not receive a transplant (Figure 3D, black bars) and similar to mice that received an LPS-loaded implant (Figure 3D, red bars). Of CB recipients, the nMIC-DiD signal was significantly higher in the CB graft compared to all other organs except the lungs and the liver, indicating that nMIC can indeed target the inflamed graft site after IV administration. In all mice, nMIC-DiD accumulated in lungs, again likely due to its high degree of vascularization and to the vast alveolar region where small nanoparticles can be easy absorbed from the systemic circulation through thanks to the thin layer of the epithelial cells. Moreover, the alveolar region is protected by alveolar macrophages that scavenge for foreign material along the lung surface and that can efficiently uptake our nanomicelles. At day 22, the nMIC-DiD signal in the CB site was significantly lower compared to day 1 (Figure 3E) and was not significantly different among the three different conditions (Figure 3E, blue, red, and black bars). However, nMIC-DiD fluorescence in the CB implant site (SC) was still present and was significantly higher than in all other organs, again except for the lungs, indicating that nMIC-DiD remain localized after targeting (Figure 3E, blue bars). In mice that received no implant (Figure 3E, black bars), the nMIC-DiD signal was comparable in most organs analyzed. Finally, the tendency for nMICs to accumulate first in the lungs suggests that administering nMIC at the onset of the inflammation and not prior to it could reduce off-targeted lung accumulation.

**Figure 4:**
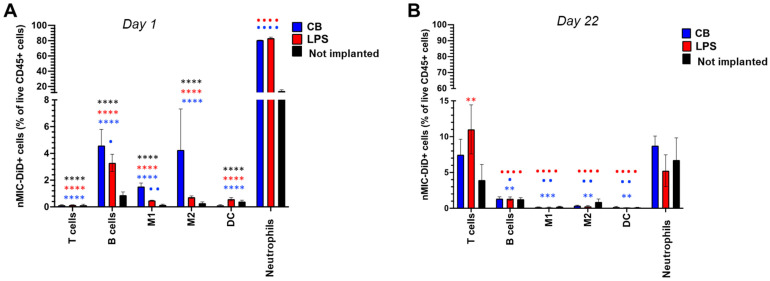
In vivo cellular uptake of nMIC-DiD in model 2 of acute inflammation. At days 1 (**A**) and 22 (**B**) post-graft incorporation, T cells (CD3^+^), B cells (CD19^+^), M1 macrophages (F4/80^+^MHC-II^+^CD206^−^), M2 macrophages (F4/80^+^MHC-II^+^CD206^+^), dendritic cells (DCs; F4/80^−^CD11c^+^), and neutrophils (CD3^−^CD19^−^Ly6G^+^) were quantified using flow cytometry for DiD-nMIC uptake in the SC graft. Data shown as percentage of live, CD45, and DiD positive cells (mean ± SD for *n* = 3 independent animals with CB + nMIC-DiD in blue, LPS + nMIC-DiD in red, and nMIC-DiD control in black). Data are expressed as mean +/− SD. A blue asterisk (*) symbol indicates a significant difference between neutrophils compared to other cells in CB + nMIC-DiD condition; a red asterisk symbol indicates a significant difference between neutrophils compared to other cells in LPS + nMIC-DiD condition; a black asterisk symbol indicates a significant difference between neutrophils compared to other cells in nMIC-DiD condition; a blue circle (•) symbol indicates a significance between T cells compared to other cells in CB + nMIC-DiD condition; a red circle symbol indicates a significance between T cells compared to other cells in LPS+DiD-nMIC condition ** *p* < 0.01, *** *p* < 0.001, **** *p* < 0.0001, as well as for circle symbols.

After harvesting and ex vivo imaging, the SC implants (CB, LPS, not implanted) were mechanically disrupted to isolate single cells for analysis using flow cytometry to quantify nMIC-DiD uptake by T cells, B cells, DCs, M1- and M2-like macrophages, and neutrophils at day 1 (Figure 4A) and 22 (Figure 4B) following IV infusion of nMIC-DiD. At day 1, neutrophils showed the highest uptake of nMIC-DiD among all immune cells. In the SC space implanted with CBs, we found that the percentage of DiD-positive B cells, M1-like macrophages, and neutrophils was higher than in the non-implanted conditions (Figure 4A, blue bars vs. black bars). In LPS-treated animals, the percentage of DiD-positive neutrophils was also higher than in the non-implanted (Figure 4A, red bars vs. black bars). The percentage of DiD-positive M2-like macrophages was higher in the CB compared to the LPS implant space (Figure 4A, blue bars vs. red bars). Although the total radiant efficiency corresponding to nMIC-DiD accumulation was overall lower on day 22 (Figure 4B) than on day 1, differences in nMIC-DiD uptake between different immune cell populations were still observed at day 22, with T cells and neutrophils showing the highest uptake of nMIC-DiD among analyzed immune cell populations in the SC CB (Figure 4B, blue bars) and LPS (Figure 4B, red bars) implant site. Thus, this study not only confirmed that nMIC can target inflammation sites, but also provided information that could be useful in developing specific immunotherapies using our DIANA. As neutrophils are first responders during inflammation, DIANA uptake by them could be particularly beneficial to deliver drugs that can decrease innate inflammation. On the other hand, immunosuppressant drugs that act on cytotoxic T cells and can decrease T-cell-mediated allograft destruction, can be delivered later, and reduce the risk of toxicity and off-targeting.

*(ii). Localized distribution and cellular uptake of DiD-labeled nFIB*

**Figure 5:**
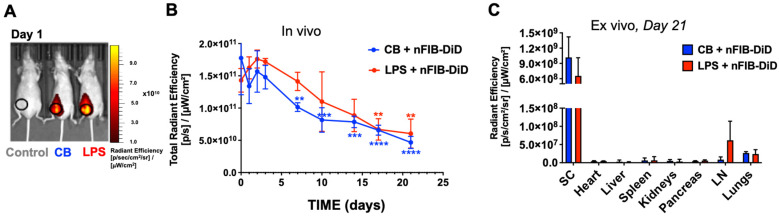
Biodistribution of nFIB-DiD in model 2 of acute inflammation. Localized acute inflammation was obtained using SC implantation of either CB (blue) or 25 μL of LPS (red) into a BSc in hairless SKH-1 mice. Five μL of nFIB-DiD was mixed with CB or LPS and implanted together in the SC BSc. (**A**) Biodistribution of nFIB-DiD co-implanted either with CB or LPS at day 1 after implantation as observed using in vivo whole-body imaging with IVIS spectrum. Radiant efficiency color scale: min = 1.0 × 10^10^, max = 1.0 × 10^11^. (**B**) Time-profile of nFIB-DiD fluorescence intensity quantified using the right inguinal area as ROI in mice with the either the CB (blue circle) or the LPS implant (red circle) from day 1 to 21. Stars indicate statistically significant differences versus time 0 in the CB and LPS implants (blue and red, respectively; two-way ANOVA with Tukey’s post hoc testing; ** *p* < 0.01, *** *p* < 0.001, **** *p* < 0.0001. (**C**) Distribution of nFIB-DiD determined ex vivo at day 21 in the SC implant site containing either CB (blue) or LPS (red) within a BSc. Explanted organs were imaged using the IVIS spectrum system and their fluorescence intensity was quantified using the Living Imaging Software and the ROI method. Total and average radiant efficiency corrected via subtraction of control background mouse (*n* = 3 independent animals; data shown as mean ± SD).

Given that the shape and length of nFIB were designed to decrease blood circulation and increase in vivo stability, we tested the efficacy of nFIB as DIANA for localized and sustained drug delivery when directly co-implanted in the site of transplantation. Five μL of nFIB-DiD were mixed with the PEG-coated beads (CB) in BSc and implanted in the SC space of SKH-1 mice. As a positive control of graft inflammation, 5 μL nFIB-DiD were also mixed with 25 μg of LPS in BSc implanted in the SC site as well in another group of SKH-1 mice. Negative control mice did not receive any implant or treatment. All mice were imaged at selected time points after transplantation using an IVIS spectrum. We found that nFIB-DiD were mainly retained in the site of implantation at day 1 (Figure 5A, black circles) and remained localized for up to 21 days (Figure 5B) in both CB and LPS implants. After explant and ex vivo IVIS imaging of the graft and selected major organs, we confirmed that the nFIB-DiD signal was present only in the SC graft tissue at day 21 post-implantation (Figure 5C).

**Figure 6:**
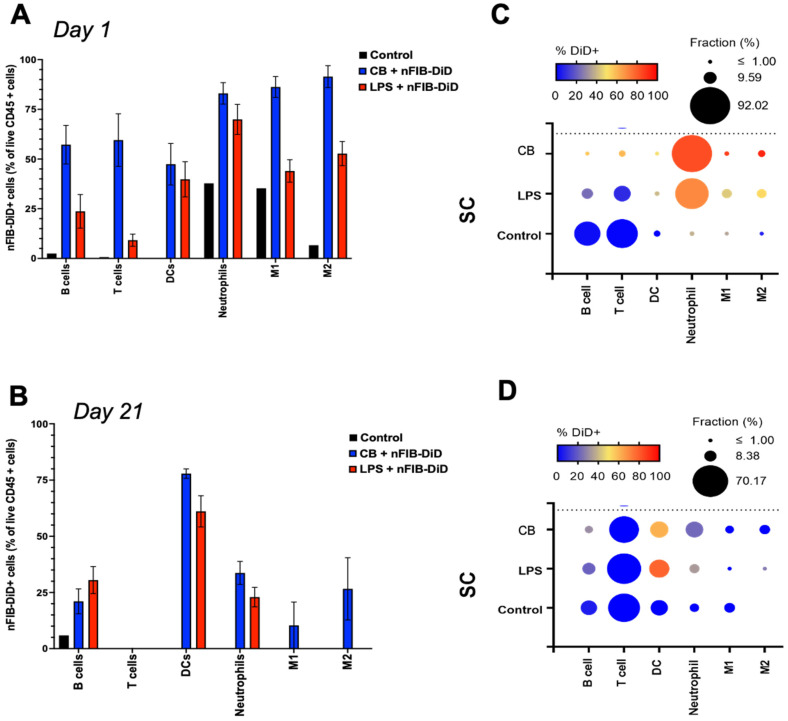
In vivo cellular uptake of nFIB-DiD in model 2 of acute inflammation. At days 1 (**A**) and 21 (**B**) post-graft incorporation, B cells (CD19^+^), T cells (CD3^+^), DCs (F4/80^−^CD11c^+^), neutrophils (CD3^−^CD19^−^Ly6G^+^), M1 macrophages (F4/80^+^MHC-II^+^CD206^−^), and M2 macrophages (F4/80^+^MHC-II^+^CD206^+^) were quantified using flow cytometry for nFIB-DiD uptake in the SC graft. In the graphs on the left (**A**,**B**), data are shown as a percentage of live, CD45^+^, and DiD positive cells (mean ± SD for *n* = 3 independent animals with CB + nFIB-DiD in blue, LPS + nFIB-DiD in red, and nFIB-DiD control in black). In the right plots (**C**,**D**), data shown are % DiD positive cells of live CD45^+^ cells (colored scale) and immune cell fraction (dot radius).

We also quantified the uptake of nFIB-DiD in different immune cell populations at days 1 (Figure 6A) and 21 (Figure 6B) in the SC implant sites. On day 1, neutrophils and macrophages primarily took up nFIB-DiD in mice bearing a CB graft, whereas at day 21, nFIB-DiD uptake shifted towards dendritic cells. These results are further highlighted in Figure 6C (day 1) and 6D (day 21) where data are shown as percentage of DiD positive cells of live CD45^+^ cells (colored scale) and immune cell fraction (dot radius). Specifically, the larger dot radius means higher fraction of immune cell sub-population present into the implant (e.g., neutrophils at day 1 are >80% in both LPS and CB implants). If more than 60% of the cell subpopulation has internalized the nFIB-DiD, the dot radius is orange (e.g., neutrophils at day 1 in the LPS implant, DCs at the 21) or red (e.g., neutrophils at day 1, CB implant). Blue and indigo dot radii indicate that instead immune cells did not internalize the nFIB-DiD or only in a very small amount, respectively (e.g., B cells and T cells at day 21).

This indicates that in the presence of local inflammation, the uptake of nFIB by neutrophils at an early stage after implantation, could benefit nFIB mediated delivery of anti-inflammatory drugs to target these innate cells that are responsible for acute inflammation at the graft site, such as that arising from biomaterial implantation.

Overall, we demonstrated that the nFIB remained localized in the SC site up to 21 days without reaching other organs in significant amounts, in agreement with our previous finding that showed how subcutaneous injection of nFIB is able to target only draining lymph nodes [14].

#### 3.3.3. Model 3: Pancreatic Islet Cell Transplantation in Mice

With the models we described above, we proved that nMIC can target an inflammation site via IV infusion and be retained at that site for several days, although accumulation also occurred within the lungs and liver. On the other hand, nFIB can be implanted in the site where an inflammation is induced, and nFIB are retained in the inflamed site without traveling into other distal sites or major organs. To improve the design of our DIANAs for future applications as localized therapies, we tested the distribution of nMIC and nFIB in a cell transplant model. We used our already established mouse transplant model [23] with islets (measured as islet cell equivalents, IEQ) implanted into the epididymal fat pad (EFP) of mice using a BSc to keep the islet graft in place and to seal the transplant site. To model syngeneic islet transplantation, we transplanted 600 IEQ islets isolated from healthy C57BL/6 mice into chemically induced diabetic C57BL/6 mice. To model allogeneic islet transplantation, we transplanted 750 IEQ islets isolated from healthy DBA/2 mice into diabetic fully MHC-mismatched C57BL/6 mice. In a pilot allogeneic study (Supporting Information, Figure S2), mice transplanted with allogeneic islets were IV infused with 50 μL of either nMIC-DiD or nFIB-DiD. IVIS analysis on the whole-body and explanted EFP graft sites revealed that nMIC but not nFIB were able to reach the implant site within 24 h of infusion (Supporting Information, Figure S2A,B). Therefore, we decided to test nFIB distribution using direct co-implantation alongside transplanted islets in the EFP of C57BL/6 mice receiving syngeneic islet transplants. We also investigated the distribution of nMIC using systemic IV administration and passive targeting in C57BL/6 mice transplanted with allogeneic islets in the EFP.

*(i) nFIB-DiR co-implantation with pancreatic islets transplanted in syngeneic mice and graft site retention*

**Figure 7:**
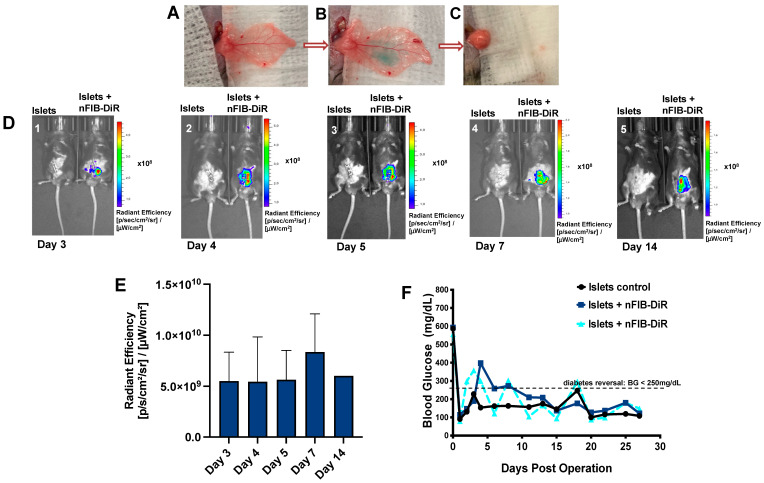
nFIB-DiR biodistribution in syngeneic pancreatic islet transplantation (model 3). (**A**–**C**) Illustration of the transplant procedure: the left gonadal fat pad was exteriorized and spread over a sterile field (**A**), islets and 15 μL of nFIB-DiR (blue) were immobilized on the EFP using a BSc (not visible) (**B**), and the EFP was folded over the islets/nFIB BSc to be placed back into the abdominal cavity (**C**). (**D**) Spatiotemporal localization of nFIB in the site of islet implantation monitored at post operatory days 3, 4, 5, 7, and 14 (panels **1** to **5**) via whole-body imaging with IVIS spectrum using the near infrared DiR fluorescence emission. Mice implanted only with islets were used as negative controls. Radiant efficiency color scale: min = 0.8 × 10^8^, max = 5.0 × 10^8^. (**E**) ROI analysis of the implanted EFPs at the different time points normalized with the background (*p* value > 0.05, *n* = 2 independent animals; data shown as mean ± SD). (**F**) Functionality of the transplanted islets as monitored via blood glucose level shows no impairment in the presence of nFIB-DiR (blue and cyan vs. black lines) indicating lack of toxicity (*n* = 2 independent animals).

The physicochemical properties of nFIB are advantageous for drug delivery via implantation or local injection rather than via systemic administration (IV). Their length (>1 μm) provides a large surface for contact with cells and tissues and their chemical composition, which includes bio-reactive groups, particularly thiols groups, on the OES chains, and allows for the interaction with proteins and biomolecules such as the BSc made of plasma-thrombin. In our transplant model, the islets are gently distributed onto the thin layer of the EFP (Figure 7A), a setting that enables the co-implantation of nFIB alongside the islets in the EFP (Figure 7B). A BSc of autologous plasma and thrombin is added onto the islets on the EFP surface to induce gel formation. The fat pad was then folded over islets/nFIB to increase the contact of the graft to the vascularized EFP (Figure 7C), placed back to the abdominal cavity, and the incision was closed with a suture.

To assess the retention of the nFIB at the site of transplant, we implanted 15 μL of fluorescent nFIB-DiR mixed with C57BL/6 islets into the EFP of C57BL/6 recipient mice (syngeneic) (Figure 7B) and performed whole-body imaging with IVIS at defined time points after the implantation (3, 4, 5, 7, and 14 days). A mouse receiving an islet transplant without nFIB-DiR acted as control. Both the images (Figure 7D; images 1 to 5) and corresponding ROI measurements (Figure 7E) indicated that the nFIB remained confined at the site of implant for at least 14 days after implantation. Regarding ROI quantification, the fluorescence emission of the nFIB-DiR remained stable during the first 14 days, confirming, as well as for model 2, the retention and prolonged localization of nanofibrils at the graft site. Longer time points will be further investigated.

By using a syngeneic transplant setting, we could also test for any toxicity caused by direct nanomaterial (nFIB) co-implantation with islets. We assessed this by monitoring blood glucose levels daily to ensure that the nFIB did not impair the insulin production of the co-transplanted islets (Figure 7F, blue squares and cyan triangles indicate the nFIB-DiR treatment in duplicated animals; black circles indicate the control). Mice that were rendered hyperglycemic chemically (STZ injection) became normoglycemic within a few days after transplantation, indicating that the co-implanted nFIB caused no significant harm to the metabolic function of transplanted islets.

*(ii) nMIC-DiD targeted distribution into an allogeneic mouse pancreatic islet transplantation site*

**Figure 8:**
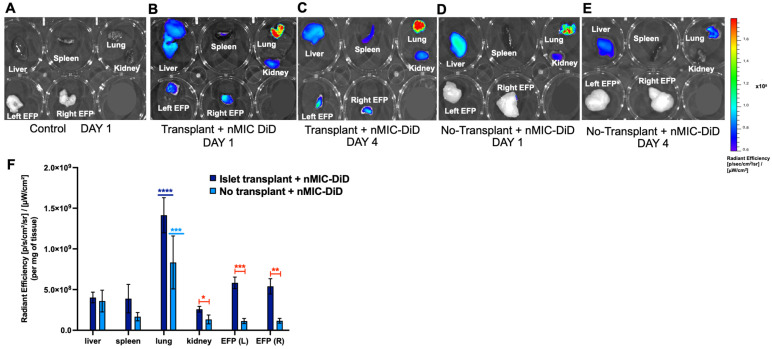
nMIC-DiD biodistribution in allogeneic pancreatic islet transplantations (model 3). C57BL/6 mice with highly compromised EFP islet transplants (nearing full rejection and no longer functional) were treated with 50 μL of nMIC-DiD via IV infusion. (**A**–**E**) Ex vivo IVIS imaging of explanted organs from control mice that were not transplanted and infused (**A**), mice bearing an islet transplant at day 1 (**B**) and 4 (**C**) after nMIC-DiD IV infusion, and mice that did not receive a transplant but received IV infusion of nMIC-DiD at day 1 (**D**) and 4 after infusion (**E**). Radiant efficiency color scale: min = 0.6 × 10^10^, max = 1.8 × 10^10^. (**F**) Quantification of DiD fluorescence of the explanted organs normalized by mass weight (mg) using ROI analysis with IVIS spectrum from transplanted and non-transplanted mice (dark and light blue bars, respectively). The asterisks in red indicate statistically significant difference between each organ of the transplanted mice and the corresponding organ of the non-transplanted mice. Dark-blue and sky-blue asterisks indicate that the presence of nMIC in the lungs is significantly higher than in all the other organs analyzed in transplanted and non-transplanted mice, respectively: * *p* < 0.05, ** *p* < 0.01, *** *p* < 0.001, **** *p* < 0.0001 (2 separate experiments with *n* = 3 animals per experiment).

To test the capability for nMIC-DiD to target and accumulate in an allogeneic islet transplant site, we transplanted islets isolated from DBA/2 mice into fully MHC-mismatched C57BL/6 recipients (allogeneic) that were rendered chemically diabetic. By daily monitoring the blood glucose level, we were able to perform IV infusion of 50 μL nMIC-DiD through the tail vein of C57BL/6 recipient mice when the islet graft was fully rejected (confirmed by blood glucose > 350 mg/dL for longer than 3 consecutive days). In such conditions, the vasculature of the EFP is likely compromised by the local inflammation driven by the graft immunogenicity, allowing for the passive targeting of EFP by nMIC. IVIS imaging of selected explanted organs was performed at day 1 (panels in Figure 8A, control animal, Figure 8B,D) and 4 (panels in Figure 8C,E, and graph in Figure 8F, dark blue) after IV infusion, which demonstrated accumulation of nMIC in the left EFP (containing the transplant) and the right EFP that did not receive a transplantation (panel in Figure 8B,C and graph in Figure 8F, dark blue). This is probably due to their vasculature being connected and often seen symmetrical response, i.e., the observation that the contralateral side of paired organs can be prominently reactive to damage [43,44]. Interestingly, in healthy mice that did not receive a transplant but received an IV infusion of nMIC-DiD, the nMIC-DiD are not present in the EFPs (panels in Figure 8D,E, and graph in Figure 8F, sky-blue). This evaluation is supported by the *p* values calculated for the difference between transplanted and non-transplanted mice (*p* = 0.0005 for the left EFPs and *p* = 0.0043 for the right EFPs), further demonstrating that the nMIC accumulate into these tissues due to passive targeting in an inflammatory setting. There was nMIC-DiD accumulation into major organs, particularly the lungs. The dark-blue asterisks (*p* value < 0.0001) and the sky-blue asterisks (*p* value = 0.0014) in the graph of Figure 8F demonstrate the presence of significantly higher amounts of nMIC-DiD in the lungs of both transplanted and non-transplanted mice, respectively, compared to all the other organs analyzed here, confirming the results observed in model 2. This unavoidable accumulation could be explained not only by the anatomy and physiology of the pulmonary circulation that receives the entire cardiac output from the right ventricle at high flow rate, but also by the fact that our nMICs are small (20–25 nm being on the smaller size of nanoparticles used for drug delivery)—much smaller than the pore size of lung fenestrae (100 nm); thus, small nMIC can easily penetrate through the lung endothelial wall [45]. Considering the versatility of our nanomaterial preparation [9,16,46], by simply changing the length and ratio of the polymeric components, we can prepare and test nMICs of various sizes and select the one that benefits the most from the enhanced vascular permeability in vivo considering that capillary leaks range in size between 24 and 60 nm [47].

In summary, nMIC DIANAs can target inflammatory sites including syngeneic and allogeneic cell transplantation sites following systemic administration; therefore, they could be used for localized delivery of therapeutic agents to such sites. Further studies are necessary to determine the optimal size of nMICs to reduce their off-target accumulation in lungs and liver and to establish their optimal dosage in allogeneic islet transplantation models.

## 4. Conclusions

Here, we investigated the biodistribution of fluorescently labeled DIANAs, in particular spherical nanomicelles (nMIC) of 20–25 nm diameter and elongated nanofibrils (nFIBs) of 5 nm diameter and >1 μm length. Results have confirmed that intravenous administration allows nFIB to only reach areas close to the infusion site that are easily accessible and well-vascularized, such as the inflamed right foot paw. After systemic intravenous administration, the circulation of nFIB is limited because their shape opposes forces to blood flow; thus, nFIB do not penetrate in sites supplied only by small capillaries (such as the inflamed EFP). Nevertheless, nFIB can be administered locally at the site of inflammation or implanted together with an islet graft without negatively impacting the transplanted cell viability and functionality. Furthermore, nFIB are retained for at least 22 days within the implant site for prolonged localized drug delivery. Therefore, future investigations will focus on using drug-loaded nFIB as localized delivery systems for anti-inflammatory treatments in the transplant site to ensure that the inflammatory response is inhibited only locally and potentially with long-lasting effects.

On the other hand, the small and spherical nMIC can circulate more effectively than nFIB, reaching any inflammation site after systemic administration (IV infusion). Thus, nMIC can passively target even sites that are confined and served by limited blood flow, such as the inflamed EFP. Our results showed that nMIC can also be retained at the inflammation site for several days. Therefore, passive targeting and retention effects are valuable properties of nMIC that already showed an ability to provide the most efficient drug loading and sustained release [14]. Here, we proved that they can be used for controlled spatiotemporal drug delivery though off-target accumulation after systemic delivery remains to be addressed by a rationale re-design of nMIC composition and delivery schedule. An advantage of our self-assembling block copolymers is that their chemistry does not need to be modified to make new nanoparticles, which can be achieved by modifying only the polymer length and mass ratio. Therefore, future work will focus on nFIB for localized delivery and nMIC for passive targeted delivery following systemic administration, and we will explore nMIC with different sizes to find those that better limit the off-target accumulation observed with the formulation that we tested in these studies.

## Data Availability

The raw data supporting the conclusions of this article will be made available by the authors on request. Data are stored and shared into and through hardware and software existing in the labs and are accessible to investigators via informal consent of the PIs. Data will be stored for at least five years after publication. Data are identified with manuscript ID, data generated and name of the PI.

## Supplementary Materials

The following supporting information can be downloaded at: https://www.mdpi.com/article/10.3390/pharmaceutics16050652/s1, Figure S1: In vivo passive targeting of LPS inflamed site by nMIC-DiD and nFIB-DiD (model 1); Figure S2: In vivo passive targeting of highly compromised EFP islet transplant site by nMIC-DiD and nFIB-DiD (model 2).
