# Supplementary material for: Drug Integrating Amphiphilic Nano-Assemblies: 2. Spatiotemporal Distribution within Inflammation Sites
Source: Pharmaceutics. 2024 May 13;16(5):652. doi: 10.3390/pharmaceutics16050652 (PMC11124943; doi:10.3390/pharmaceutics16050652)
Supplement: Supplementary file 1 [file pharmaceutics-16-00652-s001.zip › pharmaceutics-2983303-supplementary.pdf]

Drug Integrating Amphiphilic Nano-Assemblies: 2. Spatiotemporal Distribution within Inflammation Sites

Teresa De Toni [1,2,†], Teodora Dal Buono [1,†], Chris M. Li [1,3], Grisell C. Gonzalez [1], Sung-Ting Chuang [1], Peter Buchwald [1,4], Alice A. Tomei [1,2,3,5] and Diana Velluto [1,5,*]

1 Diabetes Research Institute, Miller School of Medicine, University of Miami, Miami, FL 33136, USA; detoniteresa2@gmail.com (T.D.T.); txd1299@miami.edu (T.D.B.); cml229@med.miami.edu (C.M.L.); g.gonzalez29@umiami.edu (G.C.G.); sxc2158@med.miami.edu (S.-T.C.); pbuchwald@med.miami.edu (P.B.); atomei@med.miami.edu (A.A.T.)
2 Department of Biomedical Engineering, University of Miami, Miami, FL 33146, USA
3 Department of Microbiology and Immunology, Miller School of Medicine, University of Miami, Miami, FL 33136, USA
4 Department of Molecular and Cellular Pharmacology, Miller School of Medicine, University of Miami, Miami, FL 33136, USA
5 Department of Surgery, Miller School of Medicine, University of Miami, Miami, FL 33136, USA
* Correspondence: dxv237@med.miami.edu
† These authors contributed equally to this work.

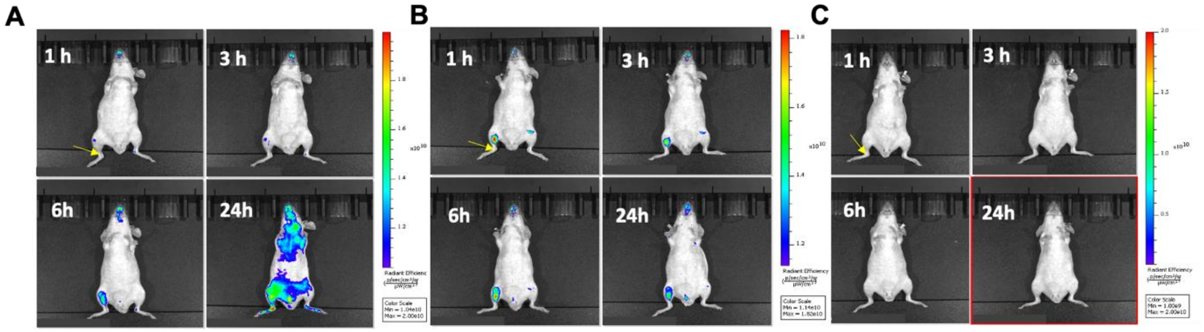

Figure S1: In vivo passive targeting of LPS inflamed site by nMIC-DiD and nFIB-DiD. SKH-1 hairless mice were treated with 25 µL of lipopolysaccharides (LPS) injected subcutaneously into the right foot paw (RFP) as indicated by the yellow arrows. Right after inducing inflammation, mice were given 50 mL of nMIC-DiD (**A**), nFIB-DiD (**B**) and free DiD (**C**) by tail vein (IV) infusion. Mice were imaged with the In Vivo Imaging System (IVIS) 1, 3, 6 and 24 hours after the IV infusions.

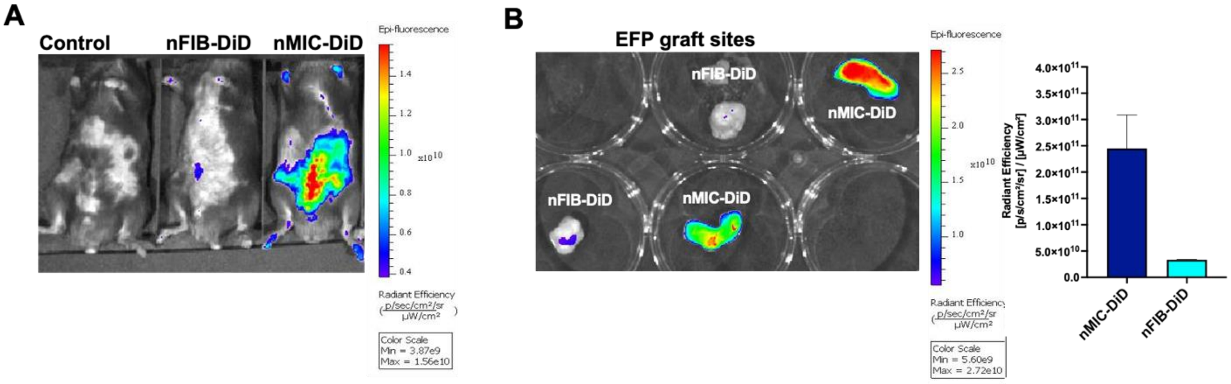

Figure S2: *In vivo* passive targeting of highly compromised EFP islet transplant site by nMIC-DiD and nFIB-DiD (model 2, pilot study). C57BL/6 mice bearing an islet graft in the EFP, were treated with 50 mL of either nMIC-DiD or nFIB-DiD via IV infusion. One day after infusion, IVIS analysis of whole-body (**A**) and ex-vivo explanted EFP organs (**B**) revealed that nFIB-DiD did not reach the implant site within 24 hours, whereas the nMIC-DiD did so efficiently. (**C**) ROI analysis of explanted EFP for mice treated with nMIC-DiD (dark blue) and nFIB-DiD (cyan). Data are mean ± SD for *n* = 2 animals.
